# Mobility-Included DNN Partition Offloading from Mobile Devices to Edge Clouds

**DOI:** 10.3390/s21010229

**Published:** 2021-01-01

**Authors:** Xianzhong Tian, Juan Zhu, Ting Xu, Yanjun Li

**Affiliations:** School of Computer Science and Technology, Zhejiang University of Technology, Hangzhou 310023, China; 2111812062@zjut.edu.cn (J.Z.); 2111812051@zjut.edu.cn (T.X.); yjli@zjut.edu.cn (Y.L.)

**Keywords:** partition offloading, deep neural networks, mobile edge computing, mobility management

## Abstract

The latest results in Deep Neural Networks (DNNs) have greatly improved the accuracy and performance of a variety of intelligent applications. However, running such computation-intensive DNN-based applications on resource-constrained mobile devices definitely leads to long latency and huge energy consumption. The traditional way is performing DNNs in the central cloud, but it requires significant amounts of data to be transferred to the cloud over the wireless network and also results in long latency. To solve this problem, offloading partial DNN computation to edge clouds has been proposed, to realize the collaborative execution between mobile devices and edge clouds. In addition, the mobility of mobile devices is easily to cause the computation offloading failure. In this paper, we develop a mobility-included DNN partition offloading algorithm (MDPO) to adapt to user’s mobility. The objective of MDPO is minimizing the total latency of completing a DNN job when the mobile user is moving. The MDPO algorithm is suitable for both DNNs with chain topology and graphic topology. We evaluate the performance of our proposed MDPO compared to local-only execution and edge-only execution, experiments show that MDPO significantly reduces the total latency and improves the performance of DNN, and MDPO can adjust well to different network conditions.

## 1. Introduction

Recently, Deep Neural Networks (DNNs) have shown their excellent advantages in some domains, such as image recognition [1], speech recognition [2] and natural language processing [3]. Technological evolution of new mobile intelligent applications goes hand-in-hand with evolution of DNNs, thus, a variety of new intelligent applications are emerging. However, running such computation-intensive DNN-based applications on resource-constrained mobile devices definitely results in long latency and huge energy consumption, which will badly affect user’s quality of service (QoS). Furthermore, the topology of DNN plays a crucial role on its accuracy and performance. The latest achievement of DNN’s topology is no longer a chain, but a directed acyclic graph (DAG). For example, the topologies of GoogLeNet [4] and ResNet [5], the champion of ImageNet Challenge in 2014 and 2015 respectively, are both DAGs. Obviously, DAG topology increases the complexity of DNN computation. DNN is so computation-intensive that user equipment (UE) only to process is not realistic, especially for its limited battery.

To solve the problem of long latency and huge energy consumption in mobile device processing, offloading the whole DNN computation to central clouds has been proposed [6]. Central clouds can provide enough computation and storage resources to UEs anytime and anywhere [7]. Although mobile device’s processing latency and energy consumption can decrease dramatically in this way, the transferring latency increases since significant amounts of data need to be transferred to the cloud over wireless network and the central clouds are always far from UEs, in terms of network topology. As a result, there still exists a big latency to process DNN jobs [8]. It’s not suitable for these services with extra low-latency requirements.

In order to reduce the total latency, transferring latency must be reduced. Thus, the computation and storage resources should be deployed at the edge of mobile network, for example, cloud servers can be integrated into base stations [9], which are called edge clouds. And UEs can offload DNN computation to edge clouds [10]. Furthermore, as the resources in edge clouds are limited and the resources in mobile devices become more powerful and energy efficient, we can leverage both resources in edge clouds and mobile devices. The layer-constituted structure of DNN is suitable for partial offloading. In a word, mobile devices process parts of DNN layers at local and edge clouds process the rest to achieve the collaborative execution. Mobile users can process these DNN layers with light computation while the edge clouds process other DNN layers with heavy computation, which can minimize the total latency and reduce the energy consumption of the mobile user [11].

Furthermore, UE’s mobility will bring some difficulties in service continuity. As we know, two obvious obstacles in 5G network are the high propagation loss and low communication distance [12]. Thus, in the coming 5G era, the deployment of base stations will be much more intensive. It will be frequent for UE to handover its serving base station when the UE is moving. When the UE moves out of the coverage region of a base station, their connection is broken, if the result generated at edge cloud hasn’t been transferred back to the UE, the UE has to process the DNN job again. If the UE is continuously moving, connection failure may happen frequently, which will seriously affect the user’s QoS [13]. A stable partition offloading decision should be made to ensure the UE’s service continuity [14].

In this paper, considering UE’s mobility included, we develop a mobility-included DNN partition offloading algorithm (MDPO) in a mobile-edge system. The objective of MDPO is minimizing the total latency by developing the optimal partition offloading decision without loss of DNN accuracy. MDPO is suitable for both DNNs with chain topology and DAG topology. MDPO is divided into two steps: (1) The first step is just for these DAG-topology DNNs, we convert the DAG topology to a chain topology by a DAG-to-Chain algorithm; (2) In the second step, we construct a DAG from a chain topology to represent the collaborative execution paths by the mobile device and the edge cloud for a DNN query at the layer level, using a Chain-to-DAG algorithm. We can find the shortest path in the new constructed DAG using a shortest path algorithm, and it’s the optimal partition offloading decision in static environment, we can easily get the optimal partition offloading decision taking the UE’s mobility into account from the shortest path.

The rest of this paper is organized as follows. In Section 2, we review some related works. Section 3 describes the system model and system architecture of a mobile-edge dynamic system. In Section 4, we explain the MDPO algorithm in detail. We evaluate our work in Section 5 and conclude in Section 6.

## 2. Related Work

MDPO is built on previous studies in edge computing. In 2009, the first edge computing concept bringing the computation storage closer to the UEs, is cloudlet [15]. Cloudlet is a decentralized system placing computers with high computation power at strategic locations in order to provide both computation and storage resources for the UEs in vicinity [16]. A more general concept of edge computing, compared to cloudlets, is fog computing. Fog computing has been introduced in 2012 to enable a processing of the applications on billions of connected devices at the edge of network [17]. Consequently, fog computing is considered as one of key enablers of Internet of Things (IoT) [18]. Another concept integrating the edge computing into the mobile network architecture is developed in 2014. The solution outlined is known as Mobile Edge Computing (MEC). In this paper, the system we discussed is a MEC system.

With the advent of MEC, a critical use case regarding the MEC is the computation offloading as this can save energy and speed up the process of computation. Lots of researches have been performed about partial computation offloading from resource-constrained mobile devices to powerful central clouds or edge clouds. Eduardo Cuervo et al. [19] proposed a system that enables energy-aware offloading of mobile code to the infrastructure, it supports fine-grained code offloading to maximize energy savings with minimal burden on the programmer. It partitions the application processing with static program analysis and dynamic profiling, and it can determine where (edge cloud or central cloud) to process functions of a program. Juan Liu et al. [20] formulated a power-constrained delay minimization problem, they use a Markov decision process approach to determine where (local or edge) to execute each data package to adapt to the channel side information. Lei Yang et al. [21] considered the partitioning of multiple users’ computations together with the scheduling of offloaded computations on the cloud resources, to achieve minimum average completion time for all the users. They model an application as a sequence of modules and determine where (client or cloud) to execute each module by solving a recursive formula. The shortest path algorithm used in MDPO is similar to the recursive algorithm proposed in [21], however, MDPO is more complex.

DNN is a common application in MEC and accelerating the DNN execution in MEC is a hot topic. Modifying DNN models is a popular approach discussed in many recent researches. Seungyeop Han et al. [22] proposed a method generating alternative DNN models for a given task to trade off accuracy and performance or energy and choosing to execute either at cloud or at local. Ehsan Variani et al. [23] proposed a deep model which become much smaller and can be processed at local at the frame-level. Ben Taylor et al. [24] proposed a strategy which allowed to select the most effective DNN from a pool of DNNs at runtime, in terms of the desired accuracy and inference time. All above papers didn’t take DNN partitioning into account at all. In this paper, we don’t change the DNN model at all, the high prediction accuracy of DNN is not sacrificed.

Recently, in order to ensure the DNN accuracy, more and more researches are focused on partial DNN computation offloading, to realize the collabrative execution of the UE and the edge clouds. NeuroSurgeon [25] investigated the compute and data characteristics of 8 DNN architectures, and proposed a light-weight scheduler to automatically partition DNN computation between mobile devices and datacenters at the granularity of DNN layers. It adapts to various DNN architectures, and chooses the partition point for best latency or best mobile energy consumption. Surat Teerapittayanon et al. [26] proposed a distributed DNN (DDNN) architecture that is over distributed computing hierarchies, consisting of central clouds, edge clouds and end devices. The objective of DDNN is reducing the communication data size among devices for the given DNN. Chuang Hu et al. [27] proposed a system partitioned DNN to be processed at both the edge cloud and the central cloud while limiting the data transmission, and it can optimally partition the DNN under the lightly loaded condition and heavily loaded condition, respectively. However, all above papers allow only fixed partitioning (partitioning the DNN into two or three partitions and processing in the order of local, edge cloud, and central cloud), while MDPO can make more flexible partitioning.

Hyuk-Jin Jeong et al. [28] took the DNN model into account and proposed a partition-based DNN offloading technique for edge computing, which divides a UE’s DNN model into a few partitions and uploads them to the edge server one by one. However, IONN is not suitable for DAG topology DNN. MDPO is suitable for both chain topology and DAG topology DNNs. Huitian Wang et al. [29] proposed an adaptive distributed DNN inference acceleration framework for edge computing environment, where DNN computation path optimization and DNN computation partition optimization are taken into consideration. Pei Ren et al. [30] proposed an edge-based collaborative object recognition solution for mobile Web AR in the 5G era, which is a differentiated DNN computation scheduling approach specially designed for the edge platform. Yinhao Huang et al. [31] proposed an offloading strategy based on Discrete Particle Swarm Optimization with Genetic Operators(DPSO-GO) to effectively reduce the cost of edge computing for offloading DNN-based applications. Zheyi Chen [32] et al. proposed a greedy and genetic algorithms based method to solve the problem of computation offloading and task scheduling for DNN-based applications in cloud-edge computing. However, all above researches do not take UE’s mobility into account, MDPO can handle the DNN computation offloading problem with mobility management in a dynamic mobile-edge system.

## 3. System Model

### 3.1. System Description

**Figure 1:**
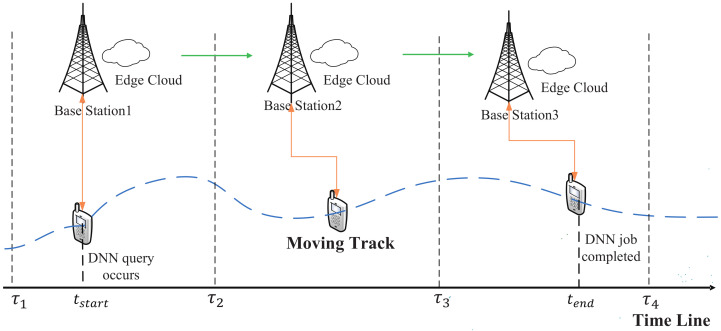
A mobile-edge dynamic system with a UE offloading DNN partition to realize the collabrative execution of mobile device and edge clouds.

In a mobile-edge network system, every base station integrates an edge cloud server. We assume that a UE is continuously moving and passing through a series of base stations. The UE’s movement pattern and base stations’ geographical distribution are known in advance. Thus, we can compute the accurate time when the UE enters the communication region of every passed base station and the time when the UE leaves. As shown in Figure 1, the UE is moving in an arbitrary route and there is a time line recording the UE’s entering time and leaving time of every passed base station. For example, the UE enters the communication region of BaseStation1 at $\tau_{1}$, and leaves the communication region of BaseStation1 at $\tau_{2}$, at the same time, the UE enters the communication region of BaseStation2, and so on.

We define $BS=\{b_{1},b_{2},\cdots ,b_{i},\cdots \}$ as the set of base stations ordered by the sequence of the UE passing by, every element $b_{i}=\left(\tau_{i},\tau_{i+1}\right)$, in which $\tau_{i}$ and $\tau_{i+1}$ denote UE’s entering time and leaving time of the *i*th base station coverage region, respectively.

With the movement of the UE, DNN query will occur at any time. The DNN job will be processed by the collaborative execution of the UE and some edge cloud servers, it may need more than one edge servers to complete because the UE maybe move fast and the time staying in one base station is too short to complete the DNN job, and these base stations can communicate with each other. We define the time DNN query occurs as $t_{start}$, the time when the DNN job is completed as $t_{end}$. We define the time that the UE spends at the same base station to process the DNN job as a time period, and the number of time periods needed to complete the DNN job as *m*. We define the set of time periods as $P=\{p_{1},p_{2},\cdots ,p_{m}\}$. The length of each time period is different. The first time period $p_{1}$ starts from the time that DNN query occurs and ends with the time that UE leaves the communication region of the base station, the last time period $p_{m}$ starts from the time that UE enters the communication region of base station and ends with the time that the DNN job is completed, other time periods between $p_{1}$ and $p_{m}$ all start from the time that the UE enters the communication region of base station and end with the time that the UE leaves the same base station. For example, if DNN query occurs at $t_{start}$ as shown in Figure 1, and the job will be completed at $t_{end}$, then the job needs three time periods to finish, i.e., m=3, the first time period is from $t_{start}$ to $\tau_{2}$, i.e., $p_{1}=\tau_{2}-t_{start}$, the second time period is from $\tau_{2}$ to $\tau_{3}$, i.e., $p_{2}=\tau_{3}-\tau_{2}$, the last time period is from $\tau_{3}$ to $t_{end}$, i.e., $p_{3}=t_{end}-\tau_{3}$.

The DNN model topology is modeled as a DAG, such as GoogLeNet shown in [4] and ResNet shown in [5]. The DNN model is embedded in the mobile device and edge clouds in advance, and the number of DNN layers is *n*. Every vertex $v_{j}$ (*j* from 1 to *n*) in DAG represents a layer of the DNN, which must be processed either at local or at edge. Our objective is to minimize the total latency of the DNN computation. Let $t_{j}^{l}$ and $t_{j}^{e}$ be the time needed to process the *j*th layer at local and at edge respectively, which can be recorded at runtime. Let $d_{j}^{t}$ denote the data size of the output of the *j*th layer. The bandwidth is defined as *B*, so we can get the transferring time of the output of the *j*th layer $t_{j}^{t}=d_{j}^{t}/B$. We define the set of above mentioned constants as $F_{l}=\left\{t_{1}^{l},t_{2}^{l},\cdots ,t_{n}^{l}\right\}$, $F_{e}=\left\{t_{1}^{e},t_{2}^{e},\cdots ,t_{n}^{e}\right\}$, $D_{t}=\left\{d_{1}^{t},d_{2}^{t},\cdots ,d_{n}^{t}\right\}$, $F_{t}=\left\{t_{1}^{t},t_{2}^{t},\cdots ,t_{n}^{t}\right\}$. In different hardware platforms, these values are different, obviously. For the same DNN job, we just need run once at a type of hardware platform to get these values.

### 3.2. System Architecture

**Figure 2:**
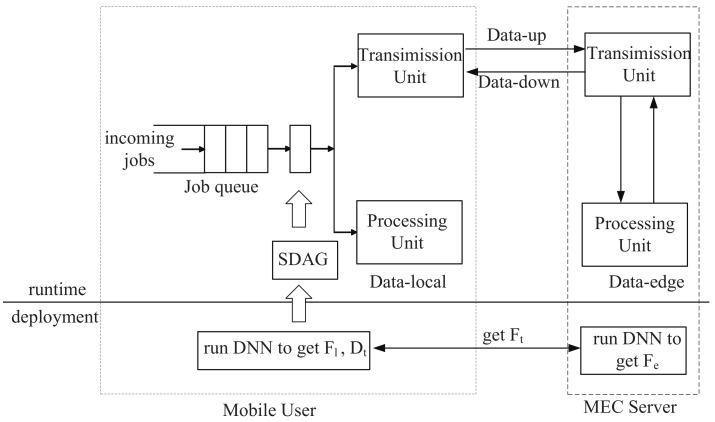
The mobile-edge partition offloading system architecture explains how the system works.

Figure 2 illustrates the overall architecture of MDPO, working in two phases. We think all MEC servers have the same computing power in the mobile-edge system. In the deployment phase, MDPO collects the execution files of DNN layers, including $F_{l}$, $F_{e}$ and $F_{t}$, and MDPO creats SDAG with these execution files. In the runtime phase, the UE can make DNN partitions according to the SDAG and complete the DNN job with MEC servers.

**Figure 3:**
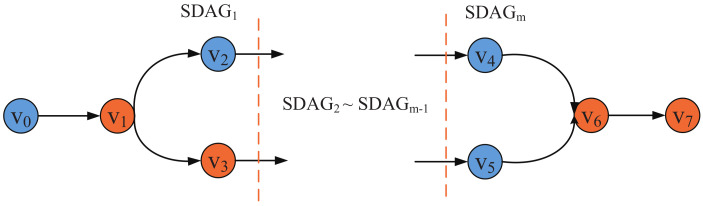
The composition of SDAG.

In details, when a DNN job being retrieved from the job queue (there may exist a waiting delay from DNN query occuring to the DNN job being retrieved from the queue, we don’t consider it in this paper), it needs *m* time periods to complete (for different DNN jobs, *m* is different and it is generated from our proposed MDPO). MDPO divides the DNN model DAG into *m* parts and only one part is processed in a time period, as shown in Figure 3. Let define the set of DNN partitions as $SDAG=\{{SDAG}_{1},{SDAG}_{2},\cdots ,{SDAG}_{m}\}$. In the $p_{i}$ time period, ${SDAG}_{i}$ is processed by the collaborative execution of UE and edge server integrated into UE’s connected base station, MDPO further divides the ${SDAG}_{i}$ into two parts, ${SDAG}_{i}^{l}$ and ${SDAG}_{i}^{e}$. ${SDAG}_{i}^{l}$ is the set of these DNN layers in ${SDAG}_{i}$ which are processed at local, such as the blue layers in Figure 3, and the ${SDAG}_{i}^{e}$ is the set of these DNN layers which are sent to the edge cloud to process, such as the orange layers in Figure 3. The layers in ${SDAG}_{i}^{l}$ and ${SDAG}_{i}^{e}$ can be discontinuous. We define these layers whose outputs need to be transferred over the network in the *i*th time period as a set $V_{i}^{t}$, for example, in ${SDAG}_{1}$, the output of $V_{0}$ should be transferred from local to edge and the output of $V_{1}$ should be transferred from edge to local. The layer output can be transferred from user to edge, from edge to user and from edge to edge. The edge clouds can transfer data to each other through the base station as shown in Figure 1. $V_{i}^{t}$ can easily get when ${SDAG}_{i}^{l}$ and ${SDAG}_{i}^{e}$ are determined.

As mentioned above, we can get the set of partitions of a DNN job as $SDAG=\{{SDAG}_{1}^{l},{SDAG}_{1}^{e},{SDAG}_{2}^{l},{SDAG}_{2}^{e},\cdots ,{SDAG}_{i}^{l},{SDAG}_{i}^{e},\cdots ,{SDAG}_{m}^{l},{SDAG}_{m}^{e}\}$. The SDAG is called the DNN execution profile, that is, the optimal partition offloading decision of the DNN. The SDAG is stored on the mobile device in the deployment phase and the user can know how to process a DNN job from the SDAG in the runtime phase. Each partition in SDAG performs its operation on the input matrice (i.e., the output matrice of the former partition ) and passes the output matrice to the next partition.

For convenience, Table 1 lists some important mathematical notations used in the paper.

## 4. MDPO for Edge Computation Offloading

### 4.1. Problem Formulation

The objective of MDPO is generating the SDAG execution profile whose total latency to process the DNN job is minimal. The total latency of DNN computation offloading can be computed as $t_{end}-t_{start}$. We can formalize the objective problem as follows:

(1) $$minimize:t_{end}-t_{start}$$

Taking UE’s mobility into account, minimizing the total latency equals to completing the DNN job with least number of passing base stations (since the time the UE spending in every base station is fixed), which also can be thought as the number of time periods, problem (1) can be equivalently re-formalized as follows:

(2) minimize:m

Furthermore, it equals to maximize the number of DNN layers to be processed at each time period. It can be realized through making the optimal decision to ensure efficient cooperation between local and edge. The total latency of the DNN job consists of three stages, which are local-processing stage, edge-processing stage and transferring stage, described as follows:

In the local-processing stage, the total local processing latency is the sum of the latency of processing every ${SDAG}_{i}^{l}$, for *i* from 1 to *m*, it can be formalized as follows:

(3) $$T_{l}=\sum_{i=1}^{m}\left(\sum_{v_{j}\in {SDAG}_{i}^{l}}t_{j}^{l}\right)$$

In the edge-processing stage, the total edge processing latency is the sum of the latency of processing every ${SDAG}_{i}^{e}$, for *i* from 1 to *m*, it can be formalized as follows:

(4) $$T_{e}=\sum_{i=1}^{m}\left(\sum_{v_{j}\in {SDAG}_{i}^{e}}t_{j}^{e}\right)$$

In the transferring stage, the outputs in the $V_{i}^{t}$ should be transferred over the wireless network, the total transferring latency can be formalized as follows:

(5) $$T_{t}=\sum_{i=1}^{m}\left(\sum_{v_{j}\in V_{i}^{t}}t_{j}^{t}\right)$$

The total latency can be calculated by adding the three stages’ latency formalized above. The objective function can be equivalently re-formalized as follows:

(6) $$minimize:T=T_{l}+T_{e}+T_{t}$$

We should guarantee that in every time period, the processing time in this time period should be less than user’s dwelling time in the base station. In the *i*th time period, which can be formalized as follows:

(7) $$\sum_{v_{j}\in {SDAG}_{i}^{l}}t_{j}^{l}+\sum_{v_{j}\in {SDAG}_{i}^{e}}t_{j}^{e}+\sum_{v_{j}\in V_{i}^{t}}t_{j}^{t}\leq p_{i}$$

Since the DNN topology is complex, we can not get the SDAG directly. Based on the objective function, we develop a mobility-included DNN partition offloading algorithm (MDPO) to generate the SDAG. The MDPO algorithm is divided into two steps to complete, for a DAG topology DNN. Otherwise, for a chain topology DNN, just step (2) can generate the SDAG. The two steps are summarized as follows: (1) The first step is just for these DAG topology DNNs, we convert the DAG topology to a chain topology by a DAG-to-Chain algorithm, this problem can be solved as a min-cut problem; (2) In the second step, we reconstruct a DAG to represent the collaborative execution paths by the UE and the edge clouds for a DNN query at the layer level, using a Chain-to-DAG algorithm. Finally, We can find the shortest path in the new constructed DAG, and we can get the SDAG easily from the shortest path.

### 4.2. DAG-to-Chain Algorithm

**Figure 4:**
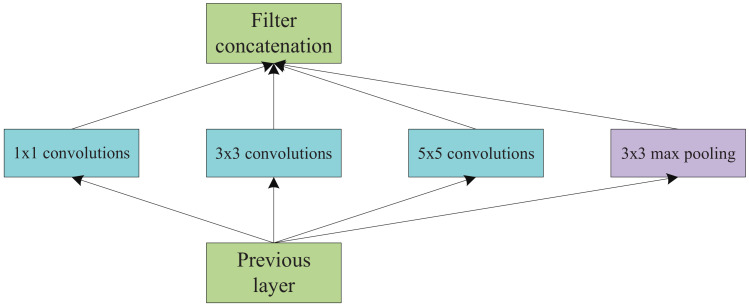
Inception module, naive version.

In this section, we introduce the first step of MDPO, i.e, the DAG-to-Chain Algorithm. By observing the topologies of lots of DNN models, we found that most DNNs are chain topology, even for these DAG topology DNNs, the overall structure is still a long chain, only some vertices have more than one branches. The most commonly used multi-branch structure is inception, such as in GoogLeNet [4]. Inception v1 is shown in Figure 4.

Based on this discovery, to simplify the partition offloading decision making, we first convert the DAG topology to a chain topology before generating the SDAG. At first, we put all multi-branch structures of the DAG in a set $Bran=\{{br}_{1},{br}_{2},\cdots ,{br}_{i},\cdots \}$. A branch structure ${br}_{i}$ is defined as the set of vertices from the beginning vertex of the branch to the branch’s convergence vertex, such as inception structure. We convert every branch to one layer, therefore, the DAG topology DNN can convert to a chain topology DNN. In order to complete the structural conversion, we need get the minimal latency of computing every ${br}_{i}$.

**Figure 5:**
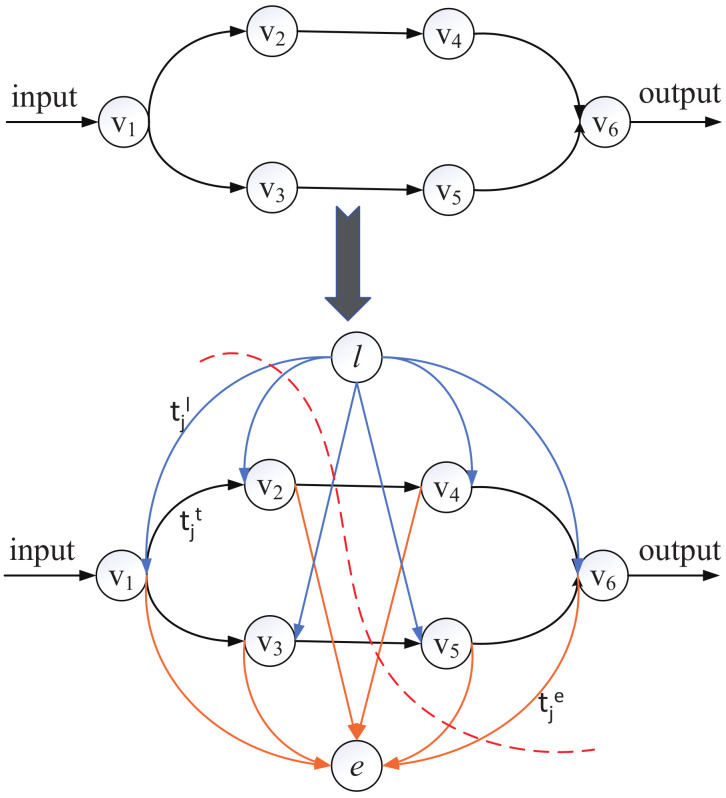
The procedure of constructing a DAG $g_{i}$ from a branch ${br}_{i}$.

The objective in this phase is to minimize the total latency of computing every ${br}_{i}$ by the the collaborative execution of the UE and the edge cloud. As the multi-branch structure always has a few layers, and the bottleneck of latency is transmission latency, we should avoid too much data transmission over the wireless network, so we just cut the branch once to realize the collaborative execution, it can be resolved as a min-cut problem. We cut the ${br}_{i}$ into two disjoint subgraphs, the former subgraph is processed at local and the latter one is offloaded to the edge cloud. The total latency consists of three stages, as formalized in Equation (6). To get the minimal latency, we construct a new graph $g_{i}$ to represent the total latency of these three stages of ${br}_{i}$ [27], so that each edge only captures a single delay value. *e* and *l* are two new vertices we add to the graph to represent the edge-processing and local-processing. The procedure from ${br}_{i}$ to $g_{i}$ is shown as Figure 5.

In the newly constructed graph $g_{i}$, blue links represent the local-processing stage, its weight is $t_{j}^{l}$. Orange links represent the edge-computing stage, its weight is $t_{j}^{e}$. Black links represent the transferring stage, its weight is $t_{j}^{t}$. To get the minimal latency, the problem can be thougt as a min-cut problem. Our job is to cut the graph into two disjoint subgraphs to ensure the total latency is minimal, requiring the vertices *l* and *e* in different subgraphs, such as the red dotted line in Figure 5. For every vertex $v_{j}$, if the link from *l* to $v_{j}$ is cut, the *j*th layer is processed at local, and if the link from $v_{j}$ to *e* is cut, the *j*th layer is processed at edge. Moreover, the black cut links represent the transmission latency. The total latency is the sum of the weights of all cut links. However, one vertex may have multiple successors and its transmission latency is cut multiple times, but we count in the total latency only once. Boykov’s algorithm [33] is used to solve the min-cut problem. We define the total latency of processing ${br}_{i}$ as $tm_{i}$.

**Figure 6:**
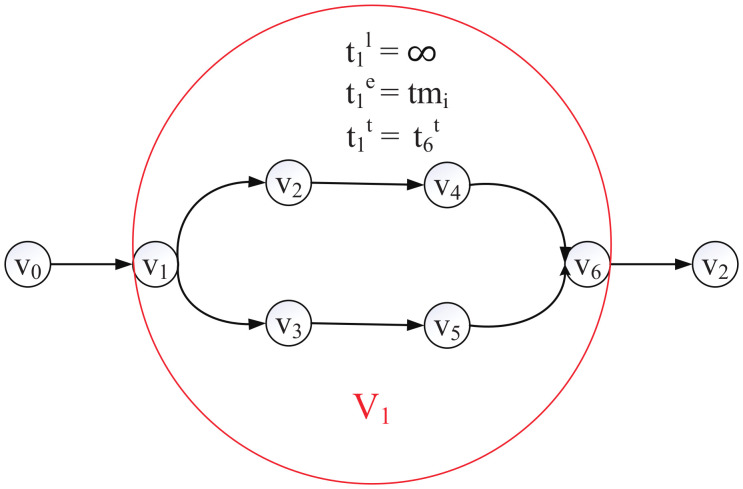
The method of converting a branch ${br}_{i}$ to a layer.

In order to convert the DAG topology to a chain topology, we merge every multi-branch structure into one layer, as shown in Figure 6. We define the local processing time of this layer as *∞* and the edge processing time of this layer as $tm_{i}$, and we renumber the layers. Then, the DAG topology DNN can convert to a chain topology DNN, defined as Chain.

The DAG-to-Chain Algorithm is concluded as Algorithm 1.
**Algorithm 1** The DAG-to-Chain AlgorithmInput: DAG, $F_{l}$, $F_{e}$, $F_{t}$Output: a newly constructed chain topology DNN Chain, $F_{l}$, $F_{e}$, $F_{t}$1 for every ${br}_{i}$ in Bran of DAG2      $g_{i}$ = new-graph-constuct( ${br}_{i}$, $F_{l}$, $F_{e}$, $F_{t}$);3      $tm_{i}=min-cut\left(g_{i}\right)$;4      $t_{i}^{l}=\infty$5      $t_{i}^{e}=tm_{i}$6 update the DAG to Chain with $t_{i}^{l}$,$t_{i}^{e}$7 update the $F_{l}$, $F_{e}$, $F_{t}$ in the Chain

### 4.3. Chain-to-DAG Algorithm

**Figure 7:**
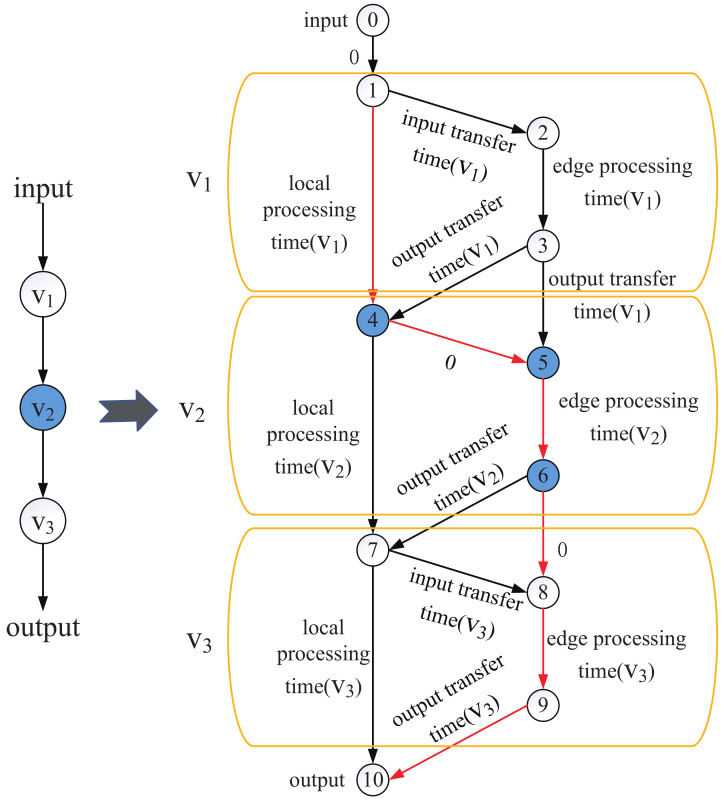
The method of converting a Chain topology DNN to a DAG topology to represent the collaborative execution paths by the UE and the edge clouds.

In this section, we introduce the second step of MDPO, i.e, the Chain-to-DAG algorithm. In this phase, our objective is to derive the SDAG to finish the DNN job with minimal latency. We construct a DAG *G* to represent the collaborative execution paths by the UE and the edge clouds for a DNN query at the layer level [28]. Figure 7 illustrates the method to create *G* from Chain, which is generated after step1. Left in Figure 6 is the chain topology DNN, and the blue vertex represents that it is converted from a multi-branch structure. We construct a DAG as shown in right. For the new constructed *G*, except for the input vertex and output vertex, each vertex in Chain is converted into three vertices. Two vertices in right belong to the edge cloud, since offloading to edge contains the edge processing time and transferring time. And one vertex in left belongs to the mobile device. Each edge is added with a weight to depict the corresponding overhead, as wrote in Figure 7. Some edges have zero weight since no computation or transmission overhead is involved.

In detail, for these layers who are not converted from a multi-branch structure like $V_{1}$, it is converted into three vertives 1, 2, and 3. The weight between 1 and 2 is the transferring time of the output of the former layer, the weight between 2 and 3 is the edge processing time of this layer, the weight between 3 and 4 is the transferring time of the output of this layer, and the weight between 1 and 4 is the local processing time of this layer. We can decide where to process this layer(local or edge) through comparing the total time of local processing and edge processing. Actually, the minimal processing time is related to the processing decision of the former layer. So we should find the best offloading decision profile all layers together.

However, for these layers who are converted from a multi-branch structure like $V_{2}$, we have decided its offloading decision in step1, the former part of the multi-branch structure is processed at local and the latter part is processed at edge. The local processing time between 4 and 7 has been valued as *∞* and the edge processing time between 5 and 6 has been valued as $tm_{2}$. So when finding the shortest path, we must choose edge processing. As the former part of the branch is processed at local, if the layer before $V_{2}$ is processed at local too, there is no transmission overhead, so the weight between 4 and 5 is 0. If the layer before $V_{2}$ is processed at edge, the output of the former layer should be transferred to mobile user, so the weight between 3 and 5 is the output transferring time of $V_{1}$. And the weight between 6 and 7 is the transferring time of the output of this layer, the weight between 6 and 8 is 0 because if the next layer is processed at edge, there is no transmission overhead.

After constructing the *G*, we can find the shortest path in *G* using shortest path algorithm, such as Dijkstra algorithm or Floyd algorithm. For example, as shown in Figure 7, we consume that the red line is the fastest execution path for a DNN query. The shortest path we found is the optimal offloading decision under the premise that the user won’t handover its connected base station, base stations’ handovering maybe bring some time loss. Therefore, the shortest path is not equivalent to the SDAG, since the UE is moving and base stations’ handovering will happen frequently. What needs to change is the execution decision of the layer which is processed at the time when the user is handovering its connected base station. In every time period, we need to satisfy the constraint as shown in Equation (7), the processing time in this time period should be less than user’s dwelling time in the base station. We can derive the SDAG from the shortest path layer by layer to satisfy the constraint.

We just need to reconfirm the execution decision of the layer which is processed at the time when the UE is handovering its connected base station. We can discuss it in two situations. (1) The first situation is that during the handovering time, the UE is doing local processing, then the UE can continue its local processing, the base station handovering has no effect on local processing. (2) The second situation is that during the handovering time the UE is doing edge processing, then the output generated at edge cannot transmit to the UE because after edge processing, the user has handovered to the next base station. In this case, the output can transfer to the next base station to continue DNN processing, but it costs a transferring time of the output. In this way, the edge processing time need to add this output transferring time between these two base stations. We can compare this new edge processing time with local processing time, and choose the way with less latency to update the SDAG.

The Chain-to-DAG Algorithm is concluded as Algorithm 2.
**Algorithm 2** The Chain-to-DAG AlgorithmInput: Chain, $F_{l}$, $F_{e}$, $F_{t}$, *P*Output: SDAG1 *G* = newgraph-constuct(Chain, $F_{l}$, $F_{e}$, $F_{t}$);2 SP = shortest-path(*G*);3 for every layer $v_{j}$ in SP4       if $v_{j}$ is processed at handovering time && $v_{j}$ is processing at edge5              $t_{j}^{e}$ += $t_{j}^{t}$;6              if $t_{j}^{e}$ > $t_{j}^{l}$7                    update SDAG by pushing $v_{j}$ to local processing set;

## 5. Evaluation

In this section, we evaluate the proposed MDPO algorithm in terms of the end-to-end latency speedup across different DNN benchmarks on different network conditions, comparing to local-only execution approach and cloud-only execution approach. (Section 5.2). We also evaluate the performance of MDPO with different moving velocities of the UE. (Section 5.3) We then compare MDPO against Neurosurgeon [25], a well-known DNN computation offloading framework. (Section 5.4)

### 5.1. Experimental Environment

We implemented MDPO on pytorch DNN framework, which is an actively developed open-source deep learning library. Our mobile device is a laptop with a Intel CPU (2.11 GHz) and 2 GB memory. Our edge server has a Intel CPU(3.60 GHz 16 cores), NVIDIA GeForce RTX 2080 Ti GPU and 64 GB memory. MDPO can make partition on both chain topology and DAG topology DNN. We evaluate the performance of MDPO on both topologies. For chain topology, AlexNet [1] and VGG16 [34] are used as benchmarks, and for DAG topology, GoogLeNet [4], ResNet [5] and MobileNet [35] are well-known models used as benchmarks. The multi-branch structure in GoogeLeNet is Inception, the multi-branch structure in ResNet is residuals module and the multi-branch structure in MobileNet is inverted residuals module. The details of these DNNs are shown in Table 2.

We consider the scenario that the geographical distribution of base stations follows an independent uniform distribution, and the radius of communication region of every base station is set as 50 m. The UE performs a uniform linear centripetalmotion with different velocities. We evaluate the MDPO performance on four kinds of network conditions, which are 3G (1 Mbps), 4G (50 Mbps), WiFi (100 Mbps) and 5G (500 Mbps). We compare the performance of MDPO with local-only execution and edge-only execution. In the edge-only execution, the UE transfers the raw input to the edge cloud, the DNN model is stored in edge cloud in advance. So the edge cloud can process the DNN job to get the final output, then the edge cloud transfer the result to the user. The edge-processing time is formalized as the sum of these three stages’ time.

### 5.2. Latency Improvement

**Figure 8:**
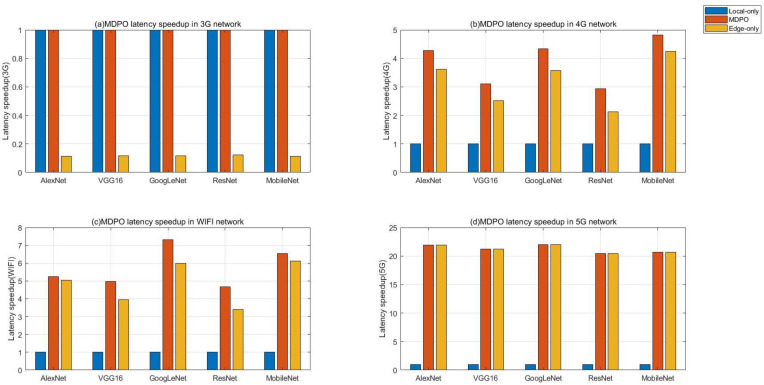
The latency speedup of MDPO across 5 DNNs on four kinds of network conditions [3G (1 Mbps), 4G (50 Mbps), WiFi (100 Mbps) and 5G (500 Mbps)] compared to local-only execution and edge-only execution. The result is normalized to local-only execution.

In this section, we evaluate the end-to-end latency speedup across 5 DNN benchmarks as shown in Table 2 on four different network conditions using MDPO, compared to local-only processing and edge-only processing, the result is shown in Figure 8. In this experiment, the velocity of mobile user is 100m/s and we use the same input for every DNN benchmark.

Figure 8a shows the result on 3G network, when the network’s is under heavy workload, the performance of our proposed MDPO equals to the performance of local-only execution for every DNN benchmark. Actually, MDPO choose to execute all layers at local on this network condition, since it costs too much time to upload the input or layer output to the edge cloud over wireless network, the transferring time is magnitude of local-processing time. So wireless network transferring is the bottleneck of edge-processing under poor network. Of course, if the data size of the input or some layer output is very small, then the transferring time can be decreased sharply, MDPO may choose to offload some partitions to edge clouds on 3G network. However, the data size of the input image in our experiment is about 600 KB and in the current Internet environment, pictures with very small size are not commonly used.

In contrary, as shown in Figure 8d, we can find that under light-workload network condition 5G, it costs little time to upload the DNN input to edge servers, and the performance of edge-only execution improves dramatically compared to heavy-workload network condition, such as 3G shown in Figure 8a. The MDPO equals to the edge-only execution for every DNN benchmark since the layer transferring time can be less than layer processing time at local on 5G network. Under light-workload network, the latency speedup depends on the computing power ratio of edge cloud and mobile device. Therefore, if the edge server’s load is heavy, more layers should be processed at local. In our proposed MDPO, we assume that the server resources are sufficient. We should take the edge cloud load into account in future work.

In Figure 8b,c, the workload of network is between 3G and 5G. Actually, the era of 3G has passed, and the era of 5G has not yet come. 4G and WiFi are commonly used in our daily life. We can find that MDPO works well in 4G and WiFi network. MDPO can utilize both computing resources in mobile devices and edge clouds to improve the computational efficiency. DNNs are composed of different types of DNN layers, such as convolution layers, pooling layers and fully-connected layers. Different types of DNN layers have different calculation features and data sizes, MDPO can always find the optimal partition approach to adapt to different DNN layer types and DNN topologies, including DAG topology and chain topology. MDPO always has better performance compared to local-only and edge-only processing. It achieves a latency speedup of 3.9× in 4G and 5.8× in WiFi on average over the local-only approach. In our proposed MDPO, we assume that the battery of mobile device is sufficient. We should take the mobile device battery into account in future work, if the battery is limited, more layers should be offloaded to edge clouds.

In conclusion, MDPO can adapts well to different network conditions, it always be the optimal strategy to process the DNN job compared to local-only execution and edge-only execution. When the network condition is well, MDPO choose to offload more DNN layers to edge clouds to process, and when the network condition is heavy, MDPO choose to process more DNN layers at local. In addition, MDPO can always make the optimal partition to adapt to different DNN topologies.

### 5.3. UE Velocity Variation

**Figure 9:**
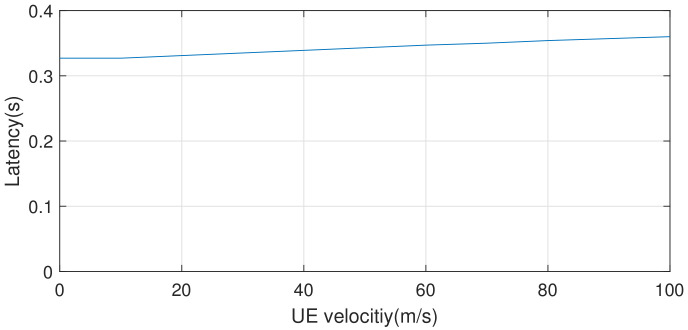
MDPO adjusts its partitioned execution as the result of varying UE Velocity.

In this section, we evaluate MDPO’s resilience to UE’s different moving velocities. We evaluate the performance with AlexNet in 4G network and the result is shown in Figure 9.

We can find that the moving velocity of the mobile user has little influence on MDPO. Actually, when the UE moves faster, handovering the connection of base stations becomes more frequent, and definitely results in more wasted time. So the UE moves faster, the latency becomes a little bigger. However, the extra delay is very small and MDPO can always choose the optimal way to complete the handovering(local-processing or edge cloud communication). So MDPO can adjust well to different UE velocities.

### 5.4. Comparing MDPO against Neurosurgeon

**Figure 10:**
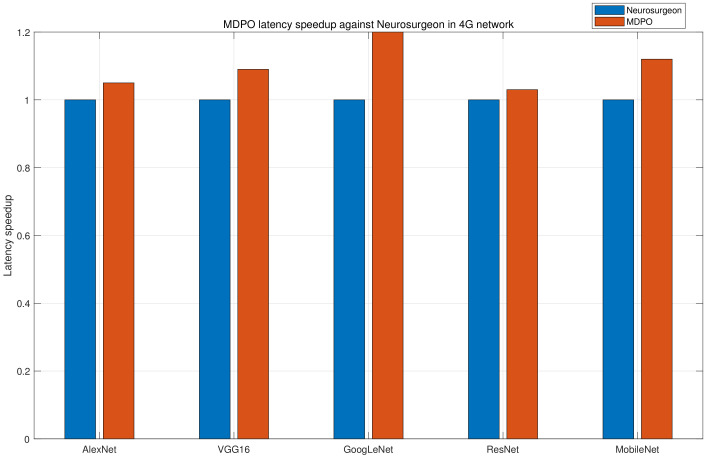
The latency speedup of MDPO against Neurosurgeon across 5 DNNs on 4G network conditions. The result is normalized to Neurosurgeon execution.

Neurosurgeon can automatically partition DNN between the mobile device and the edge cloud, but it is only effective for chain topology in static environment, while the UE won’t handover its connected base station. We compare MDPO against Neurosurgeon in a static environment, since Neurosurgeon cannot work in dynamic environment. As far as we know, MDPO is the first work taking UE’s mobility into account in DNN partitioning research. As shown in Figure 10, we can observe that MDPO outperforms Neurosurgeon because of the more flexible partition. MDPO has a latency speedup up to 1.2× and 1.1× on average against Neurosurgeon.

## 6. Conclusions

In this paper, we study DNN inference acceleration by partial offloading in MEC and propose a mobility-included DNN partition offloading algorithm, which partitions the DNN model to several partitions at layer granularity and allows the collaborative execution of the mobile user and edge clouds. It can work well in dynamic environment when the mobile user handovers its connected base station frequently. The MDPO is suitable for both DAG topology and chain topology DNNs. Experimental results show that MDPO can significantly reduce total latency and improve performance of DNN computation compared to local-only execution, edge-only execution and a well-known DNN computation offloading framework, Neurosurgeon. The moving patterns of the UE have little influence on the performance of MDPO and MDPO can adapt well to different network conditions.

## Figures and Tables

**Table 1:** Notation Description.

Notation	Definition
$BS=\{b_{1},b_{2},\cdots ,b_{i},\cdots \}$	the set of base stations
$t_{start}$	the time DNN query occurs
$t_{end}$	the time when the DNN job is completed
*m*	the number of time periods
$P=\{p_{1},p_{2},\cdots ,p_{m}\}$	the set of time periods
*n*	the number of DNN layers
$v_{j}$	a DNN layer
$F_{l}=\left\{t_{1}^{l},t_{2}^{l},\cdots ,t_{n}^{l}\right\}$	the set of DNN layer’s local processing time
$F_{e}=\left\{t_{1}^{e},t_{2}^{e},\cdots ,t_{n}^{e}\right\}$	the set of DNN layer’s edge processing time
$F_{t}=\left\{t_{1}^{t},t_{2}^{t},\cdots ,t_{n}^{t}\right\}$	the set of DNN layer’s output transfering time
$\;\;\;\;\;\;\;SDAG=\;\{{SDAG}_{1l},{SDAG}_{1e},\cdots ,{SDAG}_{ml},{SDAG}_{me}\}$	the DNN execution profile generated by MDPO
$V_{it}$	the set of DNN layers whose output need be
	transferred in network

**Table 2:** DNNs for Evaluation.

DNN Name	Number of Layers	DNN Topology
AlexNet	24	chain
VGG	42	chain
GoogLeNet	152	DAG
ResNet	245	DAG
MobileNet	110	DAG
